# Demography and Dispersal Ability of a Threatened Saproxylic Beetle: A Mark-Recapture Study of the Rosalia Longicorn (*Rosalia alpina*)

**DOI:** 10.1371/journal.pone.0021345

**Published:** 2011-06-30

**Authors:** Lukas Drag, David Hauck, Pavel Pokluda, Kamil Zimmermann, Lukas Cizek

**Affiliations:** 1 Faculty of Science, University of South Bohemia, Ceske Budejovice, Czech Republic; 2 Biology Centre ASCR, Institute of Entomology, Ceske Budejovice, Czech Republic; University of Western Ontario, Canada

## Abstract

The Rosalia longicorn or Alpine longhorn (Coleoptera: Cerambycidae) is an endangered and strictly protected icon of European saproxylic biodiversity. Despite its popularity, lack of information on its demography and mobility may compromise adoption of suitable conservation strategies. The beetle experienced marked retreat from NW part of its range; its single population survives N of the Alps and W of the Carpathians. The population inhabits several small patches of old beech forest on hill-tops of the Ralska Upland, Czech Republic. We performed mark-recapture study of the population and assessed its distribution pattern. Our results demonstrate the high mobility of the beetle, including dispersal between hills (up to 1.6 km). The system is thus interconnected; it contained ∼2000 adult beetles in 2008. Estimated population densities were high, ranging between 42 and 84 adult beetles/hectare a year. The population survives at a former military-training ground despite long-term isolation and low cover of mature beech forest (∼1%). Its survival could be attributed to lack of forestry activities between the 1950s and 1990s, slow succession preventing canopy closure and undergrowth expansion, and probably also to the distribution of habitat patches on conspicuous hill-tops. In order to increase chances of the population for long term survival, we propose to stop clear-cuts of old beech forests, increase semi-open beech woodlands in areas currently covered by conifer plantations and active habitat management at inhabited sites and their wider environs.

## Introduction

Organisms depending on dead-wood are among the most rapidly declining elements of European biodiversity, and thus attain a prominent position in most national red-lists of European countries [1]–[3]. Habitat loss, including low volume of dead-wood [4]–[6], and insufficient numbers of old and/or sun exposed trees [7]–[9] brought by modern forestry practices and abandonment of traditional forest management are considered among the major causes of the decline. Resulting habitat fragmentation threatens especially species with poor dispersal abilities [10], [11], as their isolated, small populations are prone to extinction as a result of environmental, demographic and genetic stochasticity [12]–[14]. Studying animal movement and demographic parameters thus become important issues in conservation biology and landscape management [15].

Large, conspicuous beetles are among the most attractive representatives of the saproxylic guild to the general public. Charismatic species, including the hermit beetle (*Osmoderma* s.l. *eremita*) (Scopoli, 1763), the stag beetle (*Lucanus cervus*) (Linnaeus, 1758) and the great capricorn beetle (*Cerambyx cerdo*) (Linnaeus, 1758) are often targeted by insect collectors and researchers. The amount of knowledge on their distribution and life history is relatively large, and they serve as model and umbrella species in biodiversity conservation [16]–[18].

The Rosalia longicorn (*Rosalia alpina*) (Linnaeus, 1758) serves as an umbrella species covering the habitat of beech forests. It is listed in the IUCN Red List of Threatened Species [19] and the EU Habitats Directive as a priority species of community interest. Its original range covers Southern and Central Europe [20], where the species has disappeared from a large part of its range [21], [22]. Only a single population survives north of the Alps and west of the Carpathians. Despite a notable decline during the last century, distribution of the species is rather continuous in both the above mountain systems [21], [23], [24]. In the south of Europe, the species is more widely distributed [25] and found also in lowlands [21], [26]. In Central Europe, the Rosalia longicorn inhabits mainly beech forests of middle and higher altitudes [21], [27], [28], although some lowland populations exist [29], [30]. The Rosalia longicorn develops in the wood of broadleaved trees, including beech, maples, elms and other genera [30]–[32]. It prefers old, sun-exposed trees in semi-open woodlands with minimum undergrowth [25]. Females oviposit into the crevices and cracks of wood. Larvae pupate in the spring; adults exit the wood through typical elliptic holes [27], [30]. Adult activity period lasts from the end of June until September. The life cycle takes at least three years [21], [28]. Some other aspects of the Rosalia longicorn biology might be inferred from information available on closely related species [33], [34].

Despite its significance, detailed knowledge on the Rosalia longicorn ecology and biology is still missing. Habitat preferences have recently been investigated [25], but data on demography, phenology and dispersal activity are still lacking. We therefore performed a mark-recapture study of the Rosalia longicorn on three hills in the Ralska Upland, Czech Republic. We estimated the size of the population, adult longevity, and dispersal ability. In order to assess reliability of mobility estimates, we studied the distribution pattern of the species on another 15 habitat patches in the Ralska Upland.

## Results

The total number of marked individuals was 595 in 2008 (Maly Bezdez – 157, Velky Bezdez – 240, Slatinne Hills – 198) and 375 in 2009 (Slatinne Hills only). The recapture rate was 26% in 2008 and 33% in 2009 (Table 1). Males were recaptured more frequently than females in both years (χ^2^ = 21.8, df = 1, p<0.001 and χ^2^ = 6.8, df = 1, p = 0.009).

**Table 1:** Summary of mark-recapture data obtained during the study of the Rosalia longicorn population in the Ralska Upland, Czech Republic.

Locality	Year	Marking period	Marking days	Marked beetles (♂/♀)	Recaptured* beetles (♂/♀)	Capture events (♂/♀)
Maly Bezdez	2008	12 Jul–7 Aug	17	96/61	45/9	173/74
Velky Bezdez	2008	16 Jul–10 Aug	19	158/82	46/7	244/95
Slatinne Hills	2008	26 Jul–8 Aug	10	122/76	35/13	219/94
Slatinne Hills	2009	5 Jul–16 Aug	39	222/153	87/36	407/209

*number of individuals caught the next day at the earliest.

In 2008, the first beetle was captured on 12 July and the last one on 10 August. In 2009, the first beetle was captured on 5 July and the last one on 16 August. Despite searches, no individuals were found before or after these periods. The length of the season was equal for both sexes. Under sunny and warm weather conditions, adult activity started in late morning (10–11 a.m.) and ceased in the late afternoon (4–6 p.m.), peaking at ∼12 a.m. and then again at ∼2 p.m.

### Demography

Based on the Jolly-Seber method, the estimated population sizes were 875 individuals (49 individuals/ha) for Maly Bezdez, 839 individuals (41 individuals/ha) for Velky Bezdez and 674 beetles (56 individuals/ha) for Slatinne Hills in 2008. The estimation for Slatinne Hills in 2009 was higher (1014 beetles, 84 individuals/ha). The results based on combined data from the three sites in 2008 gave lower estimates for both sexes but corresponding with the standard errors of estimates for individual sites. In both years, the resulting sex-ratio neared 1∶1 (Table 2).

**Table 2:** Summary of best-supported Jolly-Seber model (POPAN parametrization) used to estimate demography parameters and population sizes of the Rosalia longicorn on the studied sites in the Ralska Upland, Czech Republic.

Locality	Year	Best models	cAIC	Par.	♂♂ (±S.E.)	♀♀ (±S.E.)	Total
*Maly Bezdez*	*2008*	*ϕ(g) p(t) Pent(g) N(g)*	*624*	*23*	*366 (±115)*	*509 (±137)*	*875*
*Velky Bezdez*	*2008*	*ϕ(.) p(g+t) Pent(T^2^) N(g)*	*712*	*27*	*447 (±57)*	*392 (±76)*	*839*
*Slatinne Hills*	*2008*	*ϕ(g) p(t) Pent(T) N(g)*	*490*	*16*	*388 (±64)*	*286 (±60)*	*674*
Total*	2008	ϕ(g) p(g+t) Pent(g+T^2^) N(g)	1731	33	1096 (±107)	930 (±126)	2026
Slatinne Hills	2009	ϕ(g) p(t) Pent(T^2^) N(g)	1909	47	519 (±46)	495 (±62)	1014

Best models for all localities in both years were selected using quasi-Akaike Information Criterion (cAIC). ϕ_i_ – daily residence rate (combining mortality and emigration), p_i_ – catchability, pent_i_ – the probability of entering the population (combining natality and immigration). Parameters can be independent on sex and marking day (.), can differ between sexes (g), or can respond to time in a factorial (t), linear (T) or polynomial (T^2^) manners.

*Maly Bezdez + Velky Bezdez + Slatinne Hills 2008.

The best-fitting MARK model revealed that residence (*ϕ*) was constant in time and sex-dependent (Maly Bezdez, Slatinne Hills both years) or equal in sexes (Velky Bezdez). The catchability (*p*) was always time-dependent and equal between sexes on Maly Bezdez and Slatinne Hills in both years, and time- and sex-dependent on Velky Bezdez and for data pooled over the three sites sampled in 2008. The recruitment (*pent*) showed polynomial (T^2^) response (Slatinne Hills 2009, Velky Bezdez) and linear (T) response (Slatinne Hills 2008). It was constant in time- and sex-dependent on Maly Bezdez (Table 2).

The estimates of the Rosalia longicorn population size by Jolly-Seber method and Craig's model were similar for combined data of the three sites in 2008 (2026 individuals by Jolly-Seber, 2221 by Craig's model), and almost equal for Slatinne Hills in 2009 (1014 individuals compared to 1055).

**Figure 1:**
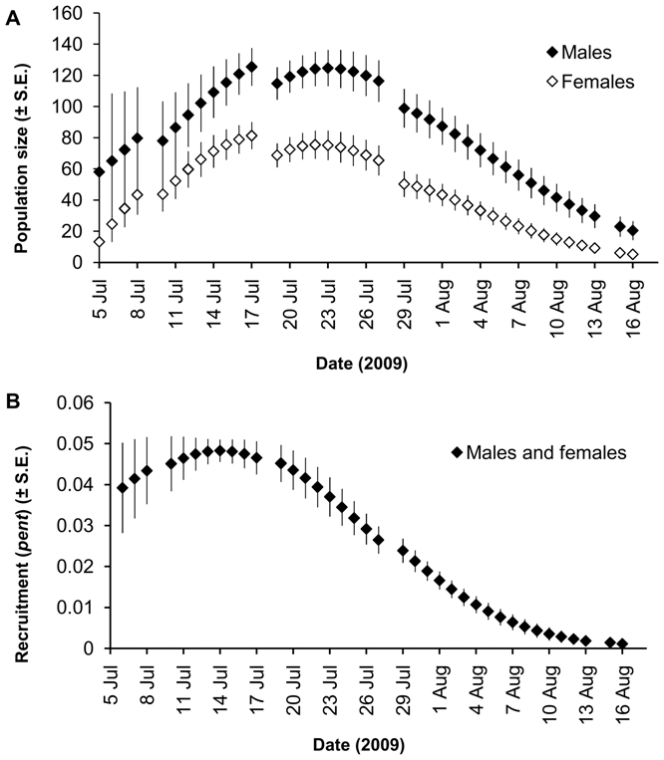
Daily estimates of population size (A) and recruitment (B) of the Rosalia longicorn in Slatinne Hills 2009. The estimates are derived from the mark-recapture data and modeled using the POPAN method in the program MARK. Used model: *(ϕ(g) p(t) Pent(T^2^) N(g).*

The daily estimates of population size were lower for females than for males; the activity patterns were synchronous for both sexes (Figure 1A). The daily estimates of recruitment (*pent*) were identical for both sexes indicating the highest rate of entering the population in the middle of July, or about a week after recording the first individual (Figure 1B).

**Figure 2:**
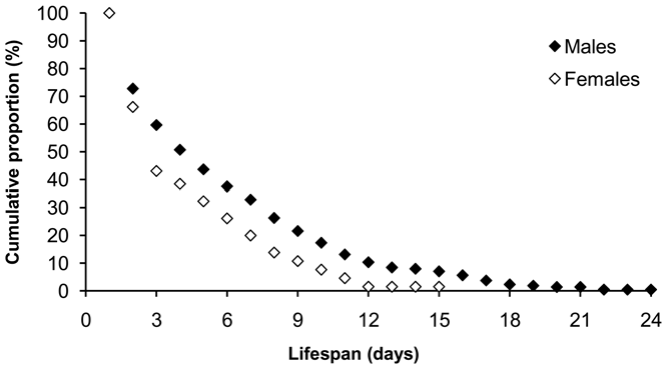
Cumulative proportions of recaptures of the Rosalia longicorn in dependence on observed “lifespan". The lifespan was counted as the number of days between the first and last capture of given individual. Data from the mark-recapture study were combined from years 2008 and 2009. (*N_males_ = 213, N_females_ = 65*).

The oldest observed male was still alive 24 days after capture; the oldest female lived for a minimum of 15 days (Figure 2). The mean residence time and its 95% C.I. based on the residence (*ϕ*; 95% C.I.), was estimated at 4.2; 3.0–6.1 days (*ϕ* = 0.79; 0.72–0.85) for females and 4.7; 3.8–5.9 days (*ϕ* = 0.81; 0.77–0.84) for males pooled over the three sites sampled in 2008. On Slatinne Hills in 2009, the mean residence time was 4.1; 3.1–5.3 days (*ϕ* = 0.78; 0.73–0.83) for females and 7.0; 5.7–8.6 days (*ϕ* = 0.87; 0.84–0.89) for males. The difference between sexes was significant only in 2009.

### Mobility and distribution

**Figure 3:**
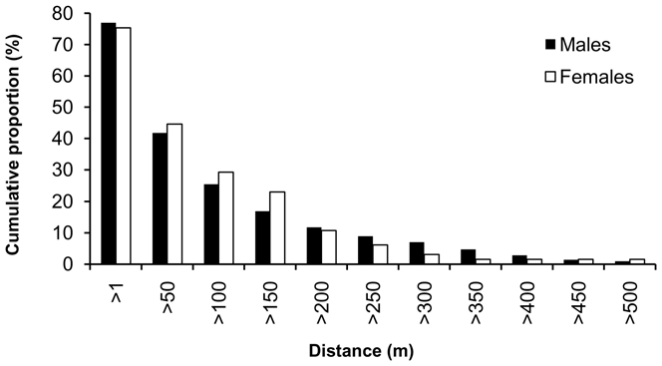
Cumulative proportion of individuals of the Rosalia longicorn in dependence on their lifetime movements. Data from the mark-recapture study were combined from years 2008 and 2009. Distances of 0 m (recaptures caught at the same place) were excluded, the rest were divided in 50 m classes (*N_males_ = 164, N_females_ = 49*).

In 2008, we recorded 93 movements of males (73.8% of recaptured beetles) and 20 movements of females (69%) between trees. In 2009 we registered 70 movements of males (80.5%) and 29 movements of females (80.6%). In both years combined, movements were recorded in 77% of recaptured males and 75% of females, and 42% of males and 45% of females moved for more than 50 m. No difference in the total dispersal distance was found between sexes (2008: Mann-Whitney U-test, p = 0.515, males: mean/median: 57/21 m, range: 0–634 m, females: mean/median: 116/25 m, range: 0–1628 m; 2009: Mann-Whitney U-test, p = 0.776, males: mean/median: 111/50 m, range: 0–658 m, females: mean/median: 86/55 m, range: 0–309 m). The longest movement was recorded in 2008, when a female was found 1628 m from original marking site (marked on Slatinne Hills and recaptured on Maly Bezdez after 11 days). The longest male movement was recorded between Maly Bezdez and Velky Bezdez (634 m) (Figure 3).

Probabilities of long-distance flights, based on the IPF regressions, estimated for males and females separately for the years 2008 and 2009 were 15–30% for 100 meters, 2.3–7.7% for 500 m, 1–4% for 1000 m, and 0.3–2.2% for 3000 m (Table 3). The fitted IPF regressions differed among the sexes neither in 2008 (slope: t = 1.330, df = 31, p = 0.097; elevation: t = −1.317, df = 31, p = 0.099) nor in 2009 (slope: t = 1.05, df = 46, p = 0.151; elevation: t = 1.121, df = 46, p = 0.134).

**Table 3:** Results of fitting the inverse power function (IPF) to movements of the Rosalia longicorn and predicted probability of movements to 100 m, 500 m, 1000 m and 3000 m.

Year	Sex	IPF: ln*I* = ln*C*(± S.E.)−*n*(±S.E.)*ln*D*	R^2^	F	df	100 (m)	500 (m)	1000 (m)	3000 (m)	Max. distance (m)
2008	M	ln*I* = −1.15(± 0.073) −4.55(±0.175)*ln*D*	0.92	248.99*	1,21	0.149	0.023	0.011	0.003	634
	F	ln*I* = −0.71(± 0.071) −3.06(±0.199)*ln*D*	0.91	99.68*	1,10	0.238	0.077	0.047	0.0218	1628
	F^a^	ln*I* = −0.86(± 0.151) −3.61(±0.443)*ln*D*	0.78	32.32**	1,9	0.195	0.049	0.027	0.0105	223
2009	M	ln*I* = −0.94(± 0.091) −3.41(±0.198)*ln*D*	0.79	107.12*	1,29	0.292	0.064	0.033	0.0117	658
	F	ln*I* = −0.85(± 0.109) −3.25(±0.276)*ln*D*	0.78	59.94*	1,17	0.272	0.07	0.039	0.0154	309

The inverse power function (IPF) expresses the probability density *I* of movements to distances *D*. The parameters *C* and *n* are estimated by fitting the logarithms of cumulative fractions of individuals moving to certain distances against logarithms of the distances.

*p<0.0001; ** p = 0.0003; ^a^ excluded max. flight (1628 m).

**Figure 4:**
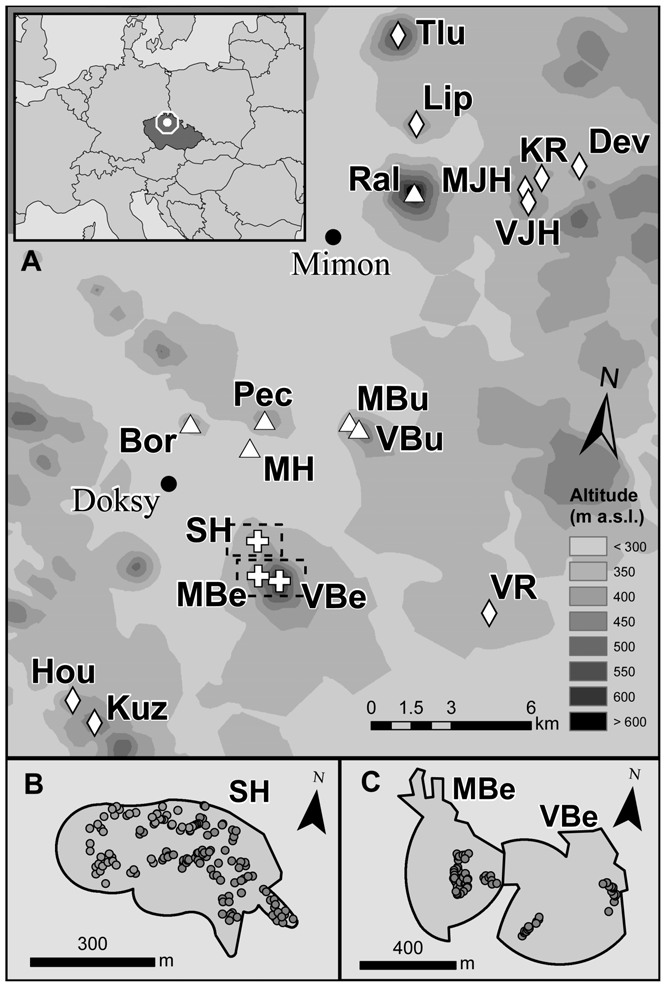
Ralska Upland, Czech Republic. Distribution of the Rosalia longicorn in the Ralska Upland (a), and the distribution of trees and dead-wood on (b) Slatinne Hills (SH), (c) Maly Bezdez (MBe) and Velky Bezdez (VBe), where the mark-recapture study took place. Eighteen sites with mature beech forest were found within the depicted area. Searching for individuals and exit holes revealed that three sites host large populations (>500 adults a year, adults regularly found and abundant: crosses), six sites host very small populations (<10 exit-holes found, adults occasionally reported: triangles), while no evidence of the beetle presence was found on the remaining nine sites (diamonds). Within the area depicted (69 100 ha), forests cover 55.5% (38 338 ha of mostly conifer plantations), mature-beech forests covered 1.1% (730.6 ha) in 2008 and 1.3% (915.6 ha) in 1953. *Abbreviations: Bor: Borny, Dev: Devin, Hou: Houska, KR: Kozi Ridge, Kuz: Kuzelik, Lip: Lipka, MBu: Mala Bukova, MBe: Maly Bezdez, MJH: Maly Jeleni Hill, MH: Mlynsky Hill, Pec: Pecopala, Ral: Ralsko, SH: Slatinne Hills, Tlu: Tlustec, VBu: Velka Bukova, VBe: Velky Bezdez, VJH: Velky Jeleni Hill, VR: Velky Radechov*.

Using aerial photos, 15 additional sites in the Ralska Upland were selected as possibly suitable for the species; a total of 18 sites with potentially suitable habitat were thus found in the region (Figure 4). Mature-beech forests covered ca. 730 ha in 2008 and ca. 916 ha in 1953; aerial photos revealed that most of the habitat loss occurred due to felling within the last 20 years, i.e. following cessation of military activities. The Rosalia longicorn individuals were found on only three sites (the same places where mark-recapture study was conducted). Single or a few exit holes were found at six further sites, indicating presence of a small population. No evidence of the Rosalia longicorn presence was discovered on the remaining nine sites (Table 4).

**Table 4:** Old beech forest patches found in the Ralska Upland, status of the Rosalia longicorn, dead-wood volume, current and historical area, altitude and geographical coordinates.

Site	*R. alpina* population	Dead-wood volume	Area (ha) 2007(1953)	Altitude (m a.s.l.)	North 50°	East 14°
Maly Bezdez	large	high	17.9 (28.8)	604	32′25″	42′49″
Velky Bezdez	large	high	20.3 (22.7)	577	32′21″	43′12″
Slatinne Hills	large	medium	12.1 (15.4)	430	33′13″	42′15″
Mlynsky Hill	small	high	11.2 (11.2)	389	34′58″	41′55″
Pecopala	small	high	202.8 (265.7)	451	35′36″	42′9″
Borny	small	medium	11.6 (19.2)	446	35′22″	39′46″
Velka Bukova	small	high	29.4 (65.2)	474	35′32″	45′20″
Mala Bukova	small	medium	14.1 (27.1)	431	35′44″	44′47″
Ralsko	small	high	217.6 (247.3)	696	40′26″	46′0″
Lipka	no evidence	low	37.9 (40.1)	473	41′42″	45′46″
Tlustec	no evidence	low	66.2 (78.7)	591	43′33″	44′39″
Devin	no evidence	medium	8.3 (8.3)	452	41′34″	51′16″
Kozi Ridge	no evidence	low	10.1 (10.1)	422	41′11″	50′8″
Maly Jeleni Hill	no evidence	medium	3.2 (3.2)	474	40′52″	49′32″
Velky Jeleni Hill	no evidence	low	19.5 (19.5)	513	40′36″	49′36″
Velky Radechov	no evidence	low	23.8 (28.5)	392	32′18″	50′7″
Houska	no evidence	medium	16.3 (16.3)	440	29′26″	37′23″
Kuzelik	no evidence	low	8.3 (8.3)	480	29′3″	38′7″

## Discussion

### Demography

Our sampling covered most of the area of studied *R. alpina* habitat patches, it spaned for the whole season, and the recapture rate was relatively high. Our data thus allow for reliable estimation of population parameters of the studied beetle. Although partly influenced by time and place, the mark-recapture approach is thus suitable for estimating population parameters of cerambycids [35].

The total population at the three studied sites (Maly Bezdez, Velky Bezdez and Slatinne Hills) consists of ∼2000 individuals a year. The life-cycle of Rosalia longicorn lasts for a minimum of three years [21]; the total number of adults that emerge during three years may reach ∼6000 individuals. The between-year fluctuations in population size are probably high as the population estimates for Slatinne Hills in 2009 were 50% higher than in 2008. Rosalia longicorn is able to reach high population densities; the adult density at the sites ranged between 41–84 adults a year per hectare of old, open, beech forest. The distribution of individuals is not even within a habitat patch [25] and the observed density is probably rather exceptionally high in comparison to other localities of the species in the Czech Republic and elsewhere [25], [36]. It might be attributed to suitable conditions at the remnants of beech forest, including large proportion of old trees and dead-wood, minimum undergrowth and open to semi-open canopy structure [25].

The mean residence time is ∼4 days for females and ∼5–7 days for males. This is short compared to maximum observed lifespan. The short mean residence time is partly attributable to reasons other than mortality such as emigration. Our results from 2008 suggest that the between sites migration is rather rare event at the study area, though. Since the whole habitat patch was thoroughly sampled on Slatinne Hills in 2009, contribution of emigration to the short residence time was probably low. We thus consider the short mean residence time as close to real lifespan of the studied beetles. Similar, though slightly longer, residence time was observed also for other longicorn species where the emigration likely played more important role [35], [37]. This suggests shorter life-span of Rosalia longicorn in comparison to other longicorns. It might be explained by the beetle biology. Numerous species of subfamily Cerambycinae, including the closely related *Rosalia coelestis*, require no food as adults [33], [38]. This very likely applies also to the studied species as no feeding was recorded during the >1500 adult capture events in this study (*pers. obs.*). Adults are active and mobile (*see below*). But since they do not feed, their energy resources are likely very limited. This may explain for the short residence time of both sexes. The shorter female residence time might be attributed to the high costs of egg production. It does not mean, though, that many females die before laying their eggs. In many Cerambycinae genera, gametogenesis is compressed into a short period in the pupal stage and imaginal gonads are senescent [38]. Females of numerous genera, including *Rosalia*, are able to copulate and oviposit nearly immediately after exiting the wood [33].

### Mobility

We found that adults frequently move among dead trees and other coarse woody debris within a habitat patch, and are able to cross considerable distances, as suggested by the observations and the predictions of long distance flight probabilities. The distribution of the Rosalia longicorn habitat is patchy and localised in the study area. The habitat spatial arrangement probably affected the estimations of the beetle mobility. Beetle movements were confined to the small habitat patches on one hand, or the beetles were forced to fly considerable distances between habitat patches. The earlier would lead to underestimation of the beetle mobility, the later would lead to its overestimation. The low number of observed long-distance movements (especially between the Maly and Velky Bezdez) indicates, though, that the vast majority of individuals remains within a few hundreds of meters of their birthplace.

Observations and dispersal ability estimates for other saproxylic beetles range from ∼200 m to ∼170 km, depending on the species, spatial scale studied and sampling method [39]. Direct comparisons of dispersal ability among studies and species are difficult. The largest movements observed are at least partly the result of small-bodied beetles' passive dispersal by wind [40]–[42]. Such passive dispersal is unlikely in large beetles, and their mobility is much lower. The longest movement of the Hermit beetle observed using mark-recapture and telemetry in Sweden did not exceed 190 m [43], [44]; whereas in France, telemetry showed movements of ∼700 m [45]. Spatial genetic structure of populations in Poland suggests average dispersal of the species at least 200 m [46]. While mark-recapture of the Stag beetle *Lucanus cervus* revealed maximum flight distance ∼150 m [47], telemetry results neared 2000 m [48]. For the darkling *Bolithophagus reticulatus*, mark-recapture study suggested limited dispersal ability [49], whereas flight-mill studies demonstrated its capability to fly for several kilometres [50] and genetic studies proposed dispersal even for tens of kilometres [51]. Mark-recapture gives lowest estimates due to underestimation of long-distance movements [52], [53]; our results thus likely underestimated the mobility of Rosalia longicorn. We may thus infer that in comparison to other large and endangered saproxylic beetles, Rosalia logicorn is highly mobile, able to actively cover distances of at least several kilometres. This is also supported by the presence of a small Rosalia population on site 10 km away from the nearest occupied sites (*see below*). Telemetric and genetic studies are needed for better understanding of its dispersal ability.

### Distribution pattern

In addition to the three “main" sites where mark-recapture was performed, signs of the species presence were found at six “minor" sites. All the “minor" sites are within ∼5 km distance from the “main" sites, except for the largest and the most conspicuous, Ralsko (Rollberg) hill, which is ∼15 km away from the “main sites" and ∼10 km from the nearest “minor sites". Occasional observations of adults and larvae by other researchers suggested the same distribution [54]–[56].

On the three “main" sites, populations consisted of hundreds of individuals a year. The presence of the species was apparent even outside the adult activity period as the typical exit holes [27] were commonly found on available dead-wood, including standing or fallen logs, broken or fallen branches of old trees (>15 cm diameter) and even relatively small logging residues on the ground. On the “minor" sites, though, the exit holes were extremely rare. They were usually localised to single trees, while most of the suitable dead-wood was unexploited. The populations at the “minor" sites are thus likely much smaller than those on “main" sites. Such small populations are prone to extinction [12]–[14]. Presence of the species on the “minor" sites is thus unlikely continuous, but it is rather a history of extinctions and re-colonization.

The species is abundant on Slatinne Hills despite the small area of the habitat (12 ha) and intensive dead-wood removal. At some of the “minor" sites, though, the conditions are at least parallel to Slatinne Hills: including terrain, volume of potentially suitable dead-wood, and extent of habitat. In comparison to Slatinne Hills, the area of old-beech forest is much larger on Pecopala and Ralsko; the dead-wood volume is larger on Mlynsky, Ralsko, and Velka Bukova hills; and finally, nearly no dead-wood removal occurs on Mlynsky and Ralsko hills owing to their conservation status. Despite the high mobility of the species and several habitat patches within its reach, the Rosalia longicorn population is concentrated on the three nearby hill-tops. The distance from the “main" sites thus seems to be the main factor affecting the Rosalia longicorn distribution in the Ralska Upland. The factors underlying such a distribution pattern require further investigation.

### Factors affecting local survival and conservation

The studied population is probably the last population of the Rosalia longicorn surviving in Central Europe north of the Alps and west of the Carpathians. It is isolated from other known populations by hundreds of kilometres, and has been probably for decades [20], [22], [30], [57].

Mature beech forests cover only a negligible portion (1.1%) of the study area (Figure 4, Table 4), which is otherwise mostly covered by conifer plantations. The extent and structure of mature-beech forests is, on the other hand, relatively stable. The study area is a former army-training ground where forestry activities were minimised between the 1950s and the 1990s, and only about 20% of the beech forest has been felled since 1953, most during the last two decades. Owing to slow succession on shallow soils of rocky slopes and hill-tops, the forest structure is also relatively stable as the abandonment of traditional managements has not yet resulted in full canopy closure and/or expansion of undergrowth at the study sites. Further, the old beech forests remained mainly on hill-tops, dominating the horizon. This possibly facilitates for effective visual location of even small habitat patches by migrating adults [58]. We consider the stability in habitat structure and the distribution pattern of habitat patches as vital factors allowing the survival of the studied population, despite the small extent of the habitat.

To increase chances for survival of the Rosalia longicorn population in the Ralska Upland, it is vital to (i) stop logging and (ii) dead-wood removal in old beech forest remnants, (iii) increase the area of semi-open beech woodlands, particularly on hill tops and slopes; and (iv) restore active management of beech pollarding/coppicing/shredding to create trees of preferred habitus.

## Materials and Methods

### Study sites

The study was carried out in the Ralska Upland (50 km north of Prague) in northern Bohemia, Czech Republic. The area is formed by sand and marlite bedrock with steep phonolite hills [59]. It is covered mainly by pine plantations with fragments of old beech forest remaining on several hill-tops. Three hills inhabited by the Rosalia longicorn were selected to carry out the mark-recapture survey, including Maly Bezdez (50°32′23.1″N, 14°42′48.4″E; 400–577 m a.s.l.; old beech forest 18 ha), Velky Bezdez (50°32′20.7″N, 14°43′11.6″E; 400–604 m a.s.l.; old beech forest 20 ha) and Slatinne Hills (50°33′13.8″N, 14°42′24.1″E; 350–430 m a.s.l.; old beech forest 12 ha). The beech forests on Maly Bezdez and Velky Bezdez are connected forming a single National Nature Reserve (28.2 ha) and Site of Community Importance (70.3 ha), with Rosalia longicorn as one of its target species. Hilltops and steeper slopes are mostly covered by low, semi-open forests with no or sparse undergrowth; even old trees are small and crooked (average DBH of the study area is 44 cm and the average height is 16 m) due to dry and shallow-soil conditions, and probably also former management. Slatinne Hills have also been declared a Site of Community Importance (138.5 ha, Rosalia longicorn as target species); the beech growth there is mainly high forest (average DBH of study area is 57 cm and average height is 27 m) on deeper soils.

Using aerial photos, other sites with old beech forests were selected within the Ralska Upland (Figure 4). Current and historical cover of old beech forest was determined for each site using the version 10 of the ArcGIS software and aerial photomaps from 1953 and 2007 [60]. Each site was inspected by experienced coleopterologists for presence of adults and exit holes for two- to six-person days, depending on the area. The search for exit holes is an effective way of locating the Rosalia longicorn populations and inhabited trees [25]; it took place in 2008, 2009 and 2010, always between 7 and 25 July, from 10 a.m. to 5 p.m., and under suitable weather conditions (*see below*). Sites were subsequently divided into three categories according to the estimated volume of available dead-wood (low, medium, high) and according to status of Rosalia longicorn local population: (i) large population – adults and exit holes commonly found, (ii) small population –exit holes and/or adults rare, (iii) no evidence – neither exit holes nor beetles observed.

Research was conducted under permit nr. 00356/KK/2008/AOPK from the Czech Agency for Nature Conservation and Landscape Protection.

### Sampling design

Mark–recapture study was conducted between 12 July and 10 August 2008 at the three sites and between 5 July and 16 August 2009 on Slatinne Hills. At each site, suitable trees (old, dead or with dead parts), coarse woody debris, and other trees (live, rotten, stumps etc.) were selected to cover as large a portion of the Rosalia longicorn habitat as possible. In Slatinne Hills, the whole area of old beech forest was covered; accessible sites with suitable trees and dead-wood were selected on Maly Bezdez and Velky Bezdez. In 2008, 59 trees were selected on Maly Bezdez, 36 on Velky Bezdez and 62 on Slatinne Hills, making total of 157 trees. In 2009, 155 trees were selected on Slatinne Hills (Figure 4B, 4C). The selected trees and coarse woody debris were searched for adult beetles in suitable weather (>15°C, no rain) between 10 a.m. and 6 p.m. All trees were numbered and visited on a regular basis; order of trees inspected was irregular.

Individuals were marked on elytra using black permanent marker, and the tip of the elytra was cut to distinguish marked individuals even if the marker was washed off. During each handling, the beetles were photographed; their body-length, sex and exact position were recorded. The individually-unique colour pattern on elytra allowed confirming each individual identity even if the marker was washed off or unreadable. Marked beetles were immediately released to their original positions. Individuals observed on the same tree more than once a day were counted only the first time. We observed no increased flight activity as a result of handling, and no flight problems due to the missing tip of elytra.

### Data analysis

The mark-recapture data were analysed in order to investigate demography and dispersal of the studied Rosalia longicorn population.

For demography analyses, we used the constrained linear models (CLM) applying the methodology of generalised linear models to mark-recapture data [61]. In MARK package [62], the Jolly-Seber method (POPAN parameterisation – suitable for open populations with births, deaths, emigration and immigration) was applied to estimate three primary parameters: *ϕ_i_* – daily residence rate (combining mortality and emigration in open populations), *p_i_* – catchability, and *pent_i_* – the probability of entering the population (combining natality and immigration). Obtained parameters are daily births (*B_i_*), daily population size (*N_i_*) and total population size (*N_tot_*). The primary parameters can be independent on sex and marking day – i.e., (.) in MARK notations, can differ between sexes (g), or can respond to time in a factorial (t), linear (T) or polynomial (T^2^) manners. Sex-time interactions can be either additive (g+t), or multiplicative (g*T^2^). From sets of models differing in parameterisation, MARK selects model(s) having high explanatory power with minimum redundant parameters, using the information theory approach (quasi-Akaike information criterion, cAIC), herein referred as best models. Best models for all localities in both years were selected. Average value of residence *ϕ*′ was obtained in MARK by defining the best-fitting models with the respective parameters not dependent on time. Comparing models where these parameters differ and not differ between sexes, i.e. *ϕ* (g) vs. *ϕ* (.), allowed direct comparison of sexes. Average residence was converted to residence time (“longevity"), using the formula -(ln *ϕ′*)^−1^ [63]. Moreover, the observed lifespan was calculated as the number of days between the first and last capture of a given individual. In order to allow comparison with results of other studies, the population sizes of males and females were also estimated for both years using the Craig's model [64].

For dispersal analyses, straight distances between capture trees were summed to obtain lifetime movements for each beetle recaptured at least once. Based on these distances, we computed for each sex the inverse power function (IPF), expressing the probability density *I* of movements to distances *D*

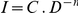



The function is fitted by plotting the logarithm of cumulative fractions of individuals moving specific or greater distances (ln*I*) against linearized expressions of the distances, i.e., ln *I*  =  ln*C* – *n*(ln*D*) [65], [66]. We compared slopes and intercepts of the resulting linear regressions using t-tests [67]. Parameter *n*, the slope of the linearized function, expresses the relative dispersal propensity, so that the shallower the slope, the higher probability of long-distance dispersal [68], [69]. We carried out these tests to compare male and female movements, and to obtain predictions of movements over long distances (100, 500, 1000 and 3000 m) within Maly Bezdez, Velky Bezdez and Slatinne Hills. Probabilities of long-distance movements, based on the IPF regressions, were estimated for males and females, separately for the years 2008 and 2009. Further, the maximum distance flight in 2008 was excluded, in order to illustrate its impact on the estimates. Due to a high number of zeroes in the data on individual movements, the nonparametric Mann-Whitney U tests were used to compare individual lifetime movements between sexes.
